# Mining basic active structures from a large-scale database

**DOI:** 10.1186/1758-2946-5-15

**Published:** 2013-03-16

**Authors:** Naoto Takada, Norihito Ohmori, Takashi Okada

**Affiliations:** 1 School of Science & Technology, Kwansei Gakuin University, 2-1 Gakuen, Sanda, Hyogo, 669-1337, Japan

## Abstract

**Background:**

The Pubchem Database is a large-scale resource for chemical information, containing millions of chemical compound activities derived by high-throughput screening (HTS). The ability to extract characteristic substructures from such enormous amounts of data is steadily growing in importance. Compounds with shared basic active structures (BASs) exhibiting G-protein coupled receptor (GPCR) activity and repeated dose toxicity have been mined from small datasets. However, the mining process employed was not applicable to large datasets owing to a large imbalance between the numbers of active and inactive compounds. In most datasets, one active compound will appear for every 1000 inactive compounds. Most mining techniques work well only when these numbers are similar.

**Results:**

This difficulty was overcome by sampling an equal number of active and inactive compounds. The sampling process was repeated to maintain the structural diversity of the inactive compounds. An interactive KNIME workflow that enabled effective sampling and data cleaning processes was created. The application of the cascade model and subsequent structural refinement yielded the BAS candidates. Repeated sampling increased the ratio of active compounds containing these substructures. Three samplings were deemed adequate to identify all of the meaningful BASs. BASs expressing similar structures were grouped to give the final set of BASs. This method was applied to HIV integrase and protease inhibitor activities in the MDL Drug Data Report (MDDR) database and to procaspase-3 activators in the PubChem BioAssay database, yielding 14, 12, and 18 BASs, respectively.

**Conclusions:**

The proposed mining scheme successfully extracted meaningful substructures from large datasets of chemical structures. The resulting BASs were deemed reasonable by an experienced medicinal chemist. The mining itself requires about 3 days to extract BASs with a given physiological activity. Thus, the method described herein is an effective way to analyze large HTS databases.

## Background

The extraction of compounds with characteristic substructures and a certain physiological activity from large chemical databases is an important step in determining structure-activity relationships. The concept of basic active structures (BASs) has been discussed previously [1]. A BAS is a substructure that is generally indicative of a certain biological activity. A set of BASs is expected to cover most of the active compounds in a given assay dataset. BASs have already been extracted for G-protein coupled receptor (GPCR)-related activity and repeated dose toxicity, and the results have been disclosed on the BASiC website [2].

Pharmaceutical companies produce in-house datasets via high-throughput screening (HTS), and these datasets can contain hundreds of thousands of compounds. The PubChem BioAssay Project releases large-scale screening databases for public use [3]. While some research has focused on predicting biological activity based on these data, the results have not provided insight on characteristic structures [4,5]. Rough set and activity landscape methods have provided useful suggestions as to the active substructure, but the number of molecules in the datasets was limited [6,7].

The extraction of BASs from these datasets provides a means of recognizing a pharmacophore with a target activity. However, the previous mining technique employed by the authors, which was based on a cascade model, was not applicable to large HTS datasets. The number of inactive compounds in such databases is usually 1000 times that of active compounds. The magnitude of this imbalance prohibits the extraction of characteristic substructures of active compounds. This difficulty is not limited to the cascade model but is also commonly encountered in most data-mining methods. The current report introduces a sampling technique that can be used to overcome the problems associated with unbalanced data. The technique uses all of the active compounds and an equal number of randomly sampled inactive compounds. Repeating the sampling process yields several sets of similar BASs while avoiding sampling biases.

The overall mining process was demonstrated by extracting BASs exhibiting HIV integrase inhibitor activity from the MDL Drug Data Report (MDDR) database. All compounds without a reference to this activity were assumed to be inactive. The tedious task of data preprocessing was reduced by the development of a KNIME workflow. The strategy was also applied to extract compounds with HIV protease activity from the MDDR database and compounds showing procaspase-3 activator activity from the PubChem BioAssay database. All of the developed software environments have been disclosed free of charge on the Internet.

## Experimental

### Workflow for pre-processing

Simple handling processes are necessary to eliminate or minimize the most tedious tasks involved in repeated sampling, data cleaning, and mining. The following section describes a KNIME (version 2.4.0) workflow that was developed to pre-process compound data [8]. The MDDR database (version 2003.1) was used as the data source targeting HIV integrase inhibitors [9]. The MDDR database contains more than 130,000 records, of which only 153 compounds show the desired activity. All other compounds were assumed to be inactive.

### Workflow

**Figure 1:**
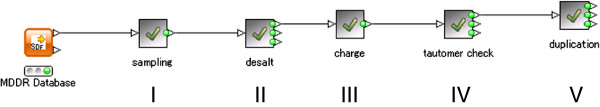
Overview of the KNIME workflow.

There are five steps in the data sampling and cleaning processes, shown as a KNIME workflow in Figure 1. Pre-processing steps are expressed as meta nodes, each of which contains several sub-workflows.

### Data sampling

**Figure 2:**
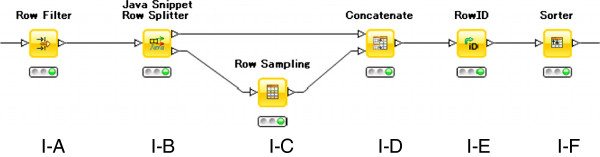
Data sampling workflow.

Meta node I contains the sampling workflow detailed in Figure 2. First, compounds with an unknown class label are filtered out in step I-A. Row splitter node I-B is used to divide the active and inactive compounds. Sampling is done in I-C and selects an equal number of inactive and active compounds. Subsequent steps assign new IDs to the concatenated table.

### Desalt

**Figure 3:**
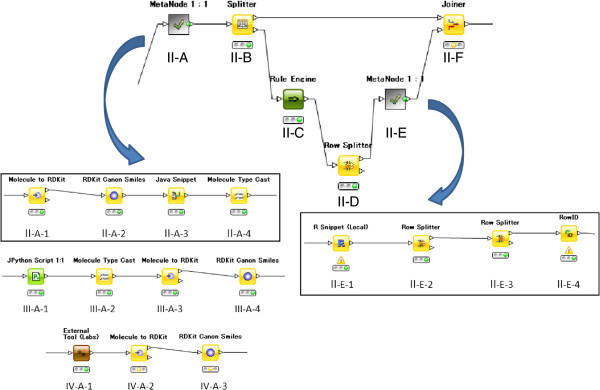
Desalt workflow.

Meta node II desalts the sampled compounds. As shown in Figure 3, meta node II-A performs the actual desalting, and II-E checks the result.

An input molecule that is converted to a molecule object of RDKit in node II-A-1 is transformed to canonical SMILES in node II-A-2. A Java program was developed to recognize and remove salts to be used in node II-A-3, and the results were converted to SMILES.

The desalt program failed when the salt was larger than the drug itself. In these cases, the meta node II-E in Figure 3 checks the results manually.

**Figure 4:**
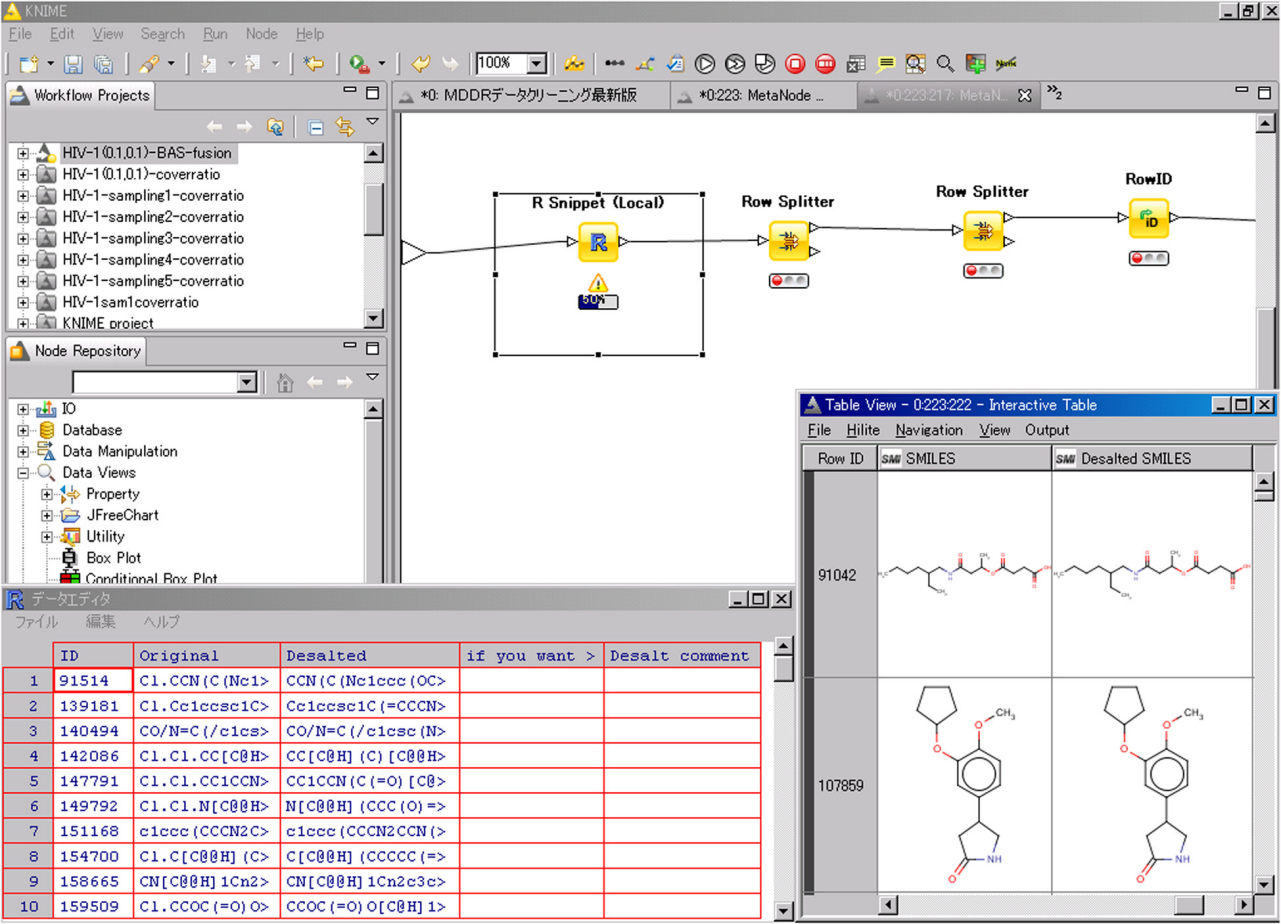
A sample screen of the interactive desalting process.

The first node II-E-1 is an R node (II-E-1), in which the R script provides an interactive editing of the SMILES string. The user interface for this step is shown in Figure 4. Candidate structures of desalted compounds can be seen at the lower right. The SMILES string in the lower left of Figure 4 is edited interactively if an erroneously desalted structure is encountered. Modified SMILES strings are then classified, and row IDs are attached in steps II-E-2, -3, and -4. In this way, interactive editing transforms the traditionally tedious desalting into an efficient process.

### Normalizing charge and tautomers

These processes use the same workflow as that shown at the top of Figure 3. Normalizing charge and tautomers use the new meta nodes III-A and IV-A depicted at the bottom of Figure 3, respectively. A Python script was developed to normalize charges in node III-A-1 of Figure 3. The following steps change the molecular format from normalized SMILES to RDKit Canon Smiles.

OpenEye’s QuacPac [10] was used to normalize tautomers in the External Tool (Labs) Node IV-A-1 in Figure 3. The following nodes serve to change the molecular format and allow interactive validation of the transformed structures. The R node is the same as that used in the desalt step.

### Removing duplicate structures

**Figure 5:**
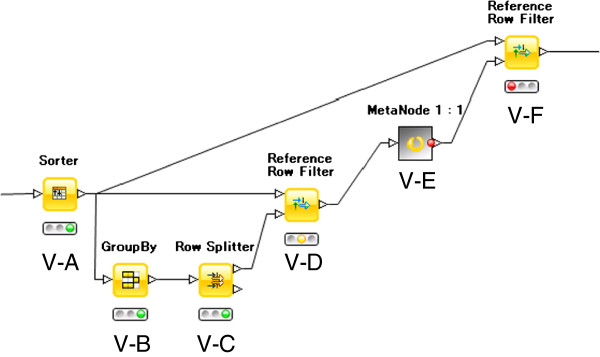
Workflow for removing duplicate compounds.

The last task in the pre-processing is the removal of duplicate compounds resulting from desalting and normalizing tautomers. The details of meta node V are shown in Figure 5, where duplicate compounds are judged using canonical SMILES and depicted on the R edit console of the V-E node (the same as node II-E). Here, compounds to be removed are selected; the others are passed on to the next step.

## Methods

The mining process was demonstrated using HIV integrase inhibitor as an example BAS activity. Three sample datasets were selected using the pre-processing technique described in the previous section. The details of the method can be found in Reference [1].

### Fragments, rules, and structural refinement

**Figure 6:**
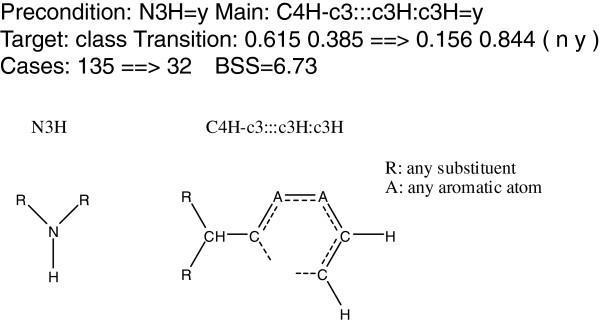
A sample rule generated by the cascade model.

BAS mining begins by the generation of linear fragments from the compound structures, each consisting of two terminal atoms and the shortest connecting path between them. In total, 192,580 fragments were generated from the three datasets. Of these, 900 fragments with a low level of correlation were selected for further analysis. The cascade model was then applied to discriminate active from inactive compounds using the linear fragments as descriptors. A brief introduction of the cascade model is provided in Additional file 1. A new implementation of the cascade model was used to generate rules using the *arules* package. This implementation provided rules defining conditions that are limited to existing fragments. That is, rules implying that some fragment does not exist were excluded. This restriction allows a wider search space. An example rule is shown in Figure 6.

This rule denotes that there are 135 compounds that meet the condition N3H=y (the existence of an amino group with at least one hydrogen atom) with an inactive/active compound ratio of 0.615/0.385. Further applying the main condition C4H-c3:::c3H:c3H=y (the existence of an aromatic ring as shown at the bottom right in Figure 6 – this includes parts of a ring or a set of fused rings) yields 32 compounds, and the ratio changes to 0.156/0.844. The last BSS denotes the strength of the rule, and its definition is given below.

Three sets of rules are obtained from three samplings using the parameters eclat_minsup = 0.1, Threshold_BSS=0.1. The total number of rules was 444. Inspection of all of the rules is time-consuming and tedious. Therefore, rules were selected using the priority measure as follows.

$$\begin{array}{lll} \;\mathrm{priority} & =\mathrm{Novelty}•\mathrm{BSS} & \\ \;\mathrm{Novelty} & =\frac{\#\mathrm{New}\_\mathrm{Covered}\_\mathrm{Compounds}\_\mathrm{by}\_\mathrm{the}\_\mathrm{Rule}}{\#\mathrm{Covered}\_\mathrm{Compounds}\_\mathrm{by}\_\mathrm{the}\_\mathrm{Rule}}\\ & \;\left(0\leq \mathrm{Novelty}\leq 1\right)\\ \;\mathrm{BSS} & =n^{A}\left\{{\left(\frac{n_{P}^{A}}{n^{A}}-\frac{n_{P}^{B}}{n^{B}}\right)}^{2}+{\left(\frac{n_{N}^{A}}{n^{A}}-\frac{n_{N}^{B}}{n^{B}}\right)}^{2}\right\} & \end{array}$$

*n*^*A*^: #Covered Compounds after the application of the main condition

*n*^*B*^: #Covered Compounds before the application of the main condition

Subscripts *P* and *N* denote positive and negative classes.

Step 1: Calculate the priority of all rules.

Step 2: Select the highest score rule.

Step 3: Repeat step 1 and step 2 among the unselected rules until priority is 0.

Here, the priority is defined as the product of *Novelty* and *BSS*. *Novelty* values are higher when the covered compounds of a rule are not covered by any previously selected rules. *BSS* (Between groups sum of squares) is the strength of a rule, and its value is higher when the number of covered compounds selected by the main condition is large and when the changes in the positive/negative ratio are large. Repetition of this rule selection scheme resulted in 55 rules being refined in the next step.

**Figure 7:**
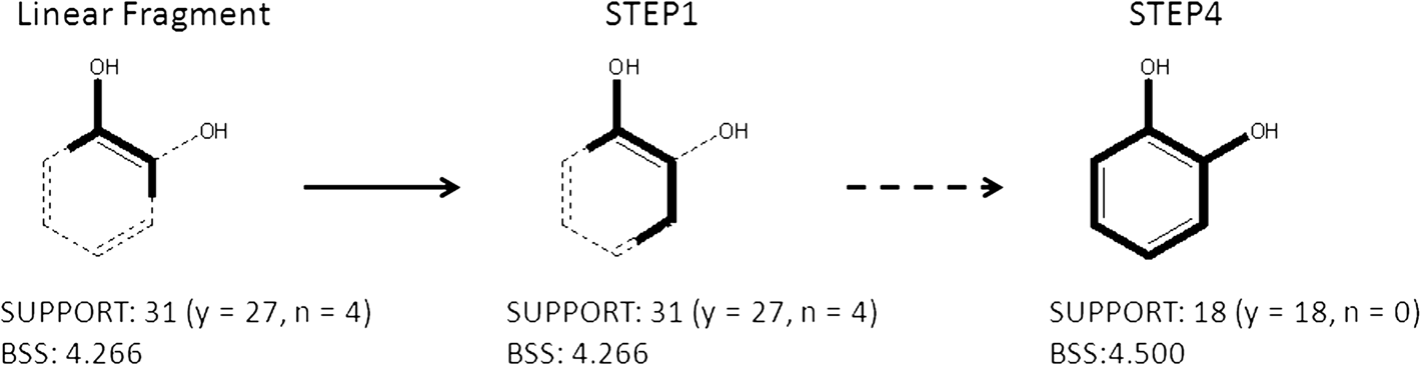
An example of structural refinement.

Structural refinement explores substructures and identifies features that discriminate active compounds from inactive compounds. An example of the refinement process is shown in Figure 7. In the example, the seed fragment HO-c:c is given by the main condition of a given rule. Here, the number of covered compounds was 31 (27 actives and 4 inactives) with an associated *BSS* value of 4.266. The refinement algorithm then re-evaluates the substructure with all possible bonds and atom pairs at all positions. The substructure with the maximum *BSS* value is employed in the next step. Step 1 in Figure 7 shows the resulting substructure HO-c:c:c. The same process is repeated until all subsequent attachments decrease the BSS value. A catechol substructure was obtained at step 4 in this example. A user with no chemical foreknowledge mechanically refined the resulting fragments in the rules to obtain BAS candidates using this refinement system. No chemical knowledge was applied to arrive at these candidates.

### Unification of BAS candidates with similar structures

**Figure 8:**
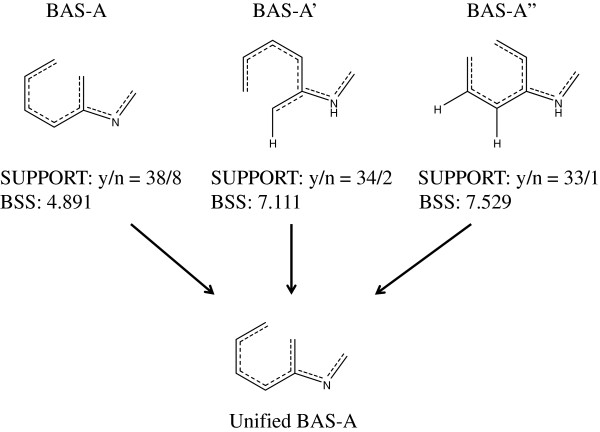
A unification example of BAS candidates.

Some candidates may seem trivial, such as a benzene ring. Usually, several of the obtained BAS candidates will be structurally similar and need to be unified. The selection and unification of BAS candidates are performed manually. Figure 8 shows an example of BAS unification of three BAS candidates. Usually, candidate structures covering the maximum number of active compounds were employed. In the given example, all active compounds of BAS-A’ and BAS-A” are contained in those of BAS-A. Therefore, BAS-A was identified as the unified BAS. If the user is an experienced chemist, the candidate expressions can be edited to yield a new BAS. A means of selecting a unified BAS is provided in the Additional file 2.

### Finding subBASs

**Figure 9:**
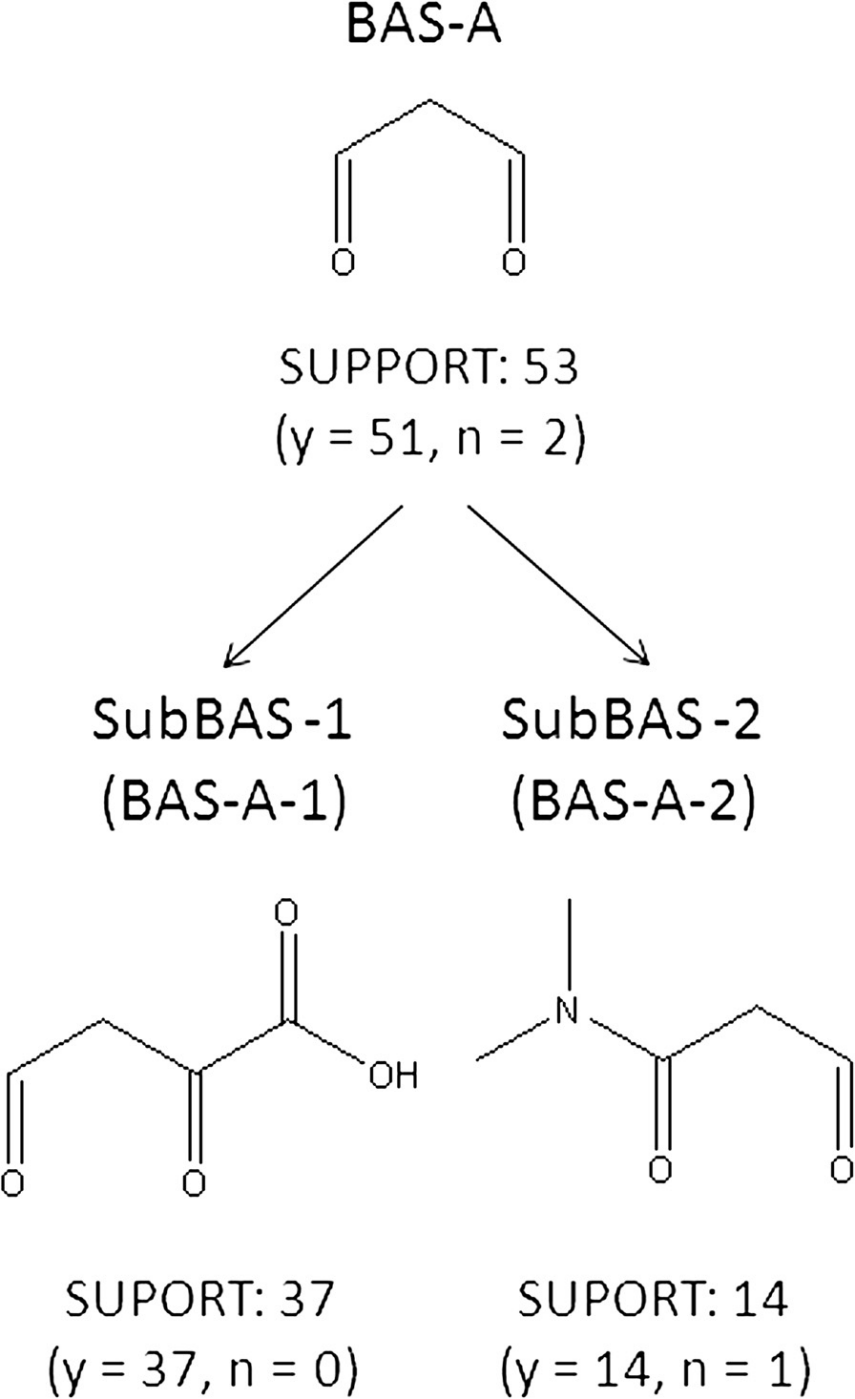
An example of subBAS extraction.

A larger substructure that is shared by a group of compounds can sometimes be identified from the covered compounds of a BAS. Such a substructure is a subBAS and is often chemically interesting. Therefore, subBASs are also registered in the knowledge base. Figure 9 shows two subBASs, BAS-A-1 and -A-2, that were identified from the covered compounds of BAS-A. The inspection of compound structures often requires chemical knowledge and understanding. The covered compounds of subBASs do not need to cover all of the compounds in the parent BAS.

### Validation of the sampling scheme

The number of samplings is critical to the success of the proposed method. Too few samplings may affect the diversity of inactive compounds, resulting in the wrong BAS structure. Too many samplings are time consuming. The cover ratio of active compounds is used to estimate the necessary number of samplings, since identified BASs should cover the majority of active compounds.

**Figure 10:**
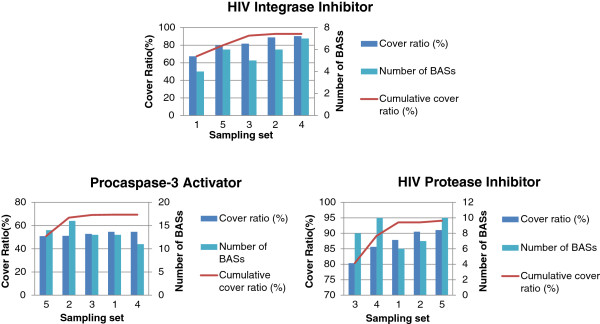
**Covering ratios and number of BASs for the sampled datasets.** Sampling sets are in the ascending order of covering ratios.

Cover ratios were examined during inactive sampling of three datasets: HIV integrase inhibitors, HIV protease inhibitors, and procaspase-3 activators. BASs were extracted from each sample dataset. The results are shown in Figure 10.

The light and dark blue bars in Figure 10 show the numbers of BASs and their cover ratios obtained from each sample dataset, respectively. Between 4 and 7 BASs were obtained, covering 65% to 88% of the active compounds in the case of HIV integrase. Thus, as expected, the BASs mined from a single sampling often fail to cover most of the active compounds. Conversely, the cumulative cover ratio, shown by the red line, reached its maximum values after three samplings in almost all cases. The saturation of the cumulative cover ratios after three samplings does not change, even after changing the sampling order of the three activities. Therefore, the estimated required number of samplings was three.

The number of final BASs obtained from the first three samplings yielded 9 BASs in the case of HIV integrase, as will be discussed in the next section. Inspection of BAS structures obtained from the 4^th^ and the 5^th^ samplings has shown that all of these BASs are contained in the final BAS sets. This was also observed with the protease and procaspase-3 activities. Therefore, three samplings were deemed sufficient to yield a stable set of BASs covering most of the active compounds.

## Results and discussion

### HIV integrase inhibitor mining results

**Figure 11:**
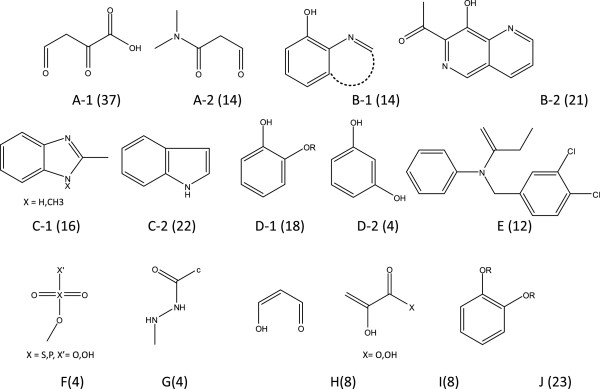
**Structures of 14 BASs obtained for HIV integrase inhibitor.** The numbers in parentheses show the number of active compounds covered by the associated BAS.

The cascade model was applied to three sample datasets as discussed in the previous section. Refinement of the 55 selected rules and subsequent BAS unification resulted in the nine BASs (A-1 to E) shown in Figure 11. Unification of similar BASs and the discarding of trivial substructures were performed without *a priori* chemical knowledge. These nine BASs covered 130 of the 153 active compounds.

**Figure 12:**
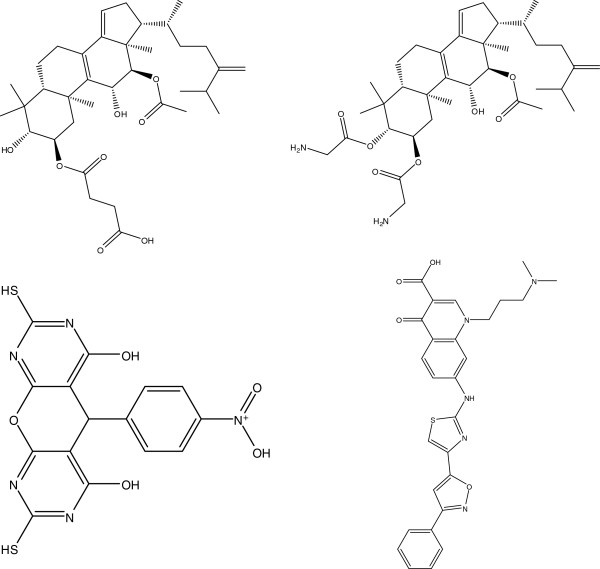
**The four compounds not covered by BASs in Figure**11**.**

This mining system cannot, however, identify characteristic substructures if their covered compounds are too few. The refinement system may still overlook meaningful BAS candidates. An experienced chemist evaluated 23 uncovered structures. While this resulted in six additional interesting substructures, one of them also appeared frequently among the inactive compounds. Finally, five new BASs were added, shown by F, G, H, I, and J in Figure 11, where BAS J is a generalization of D-1. These BASs covered 19 new compounds with only 4 of the 153 active compounds left uncovered. Structures of the four uncovered compounds are shown in Figure 12.

**Figure 13:**
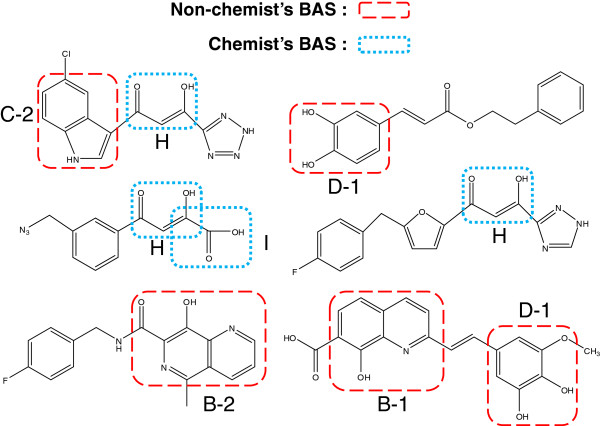
BASs in typical pharmaceuticals for HIV integrase inhibitor.

A question arises as to which components of a pharmaceutical substructure are expressed by the obtained BASs. A review paper of HIV integrase inhibitors showed seven typical active compounds [11]. The BAS components are given in Figure 13.

The red dashed lines in Figure 13 indicate BASs selected without *a priori* chemical knowledge. The blue dotted lines indicate BASs mined by an experienced chemist. This result illustrates the importance of BASs in real pharmaceuticals.

### Applications to other biological activities

#### HIV protease inhibitor

**Figure 14:**
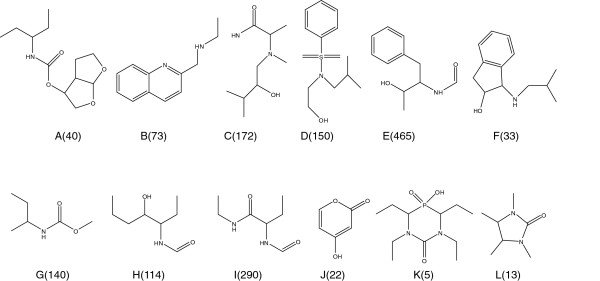
**Structures of 12 BASs for HIV protease inhibitor.** The numbers in parentheses show the number of active compounds covered by the associated BAS**.**

The next target of active compound mining was HIV protease inhibitor in the MDDR database. In total, 758 active compounds were identified, and 394 rules were obtained from three sampling datasets, each of which contained an equal number of inactive and active compounds. The rule priority criteria narrowed these to 65 rules, and subsequent structure refinement and unification resulted in the seven BASs (A to G) depicted in Figure 14. These BASs covered 78.3% of the active compounds. This ratio is lower than that obtained for HIV integrase inhibitor. An experienced chemist surveyed the structures of the 164 remaining active compounds and identified five additional BASs shown by H, I, J, K, and L in Figure 14. These covered 61 new compounds, leaving 103 uncovered compounds with miscellaneous structures.

### Procaspase-3 activators in the PubChem BioAssay database

**Figure 15:**
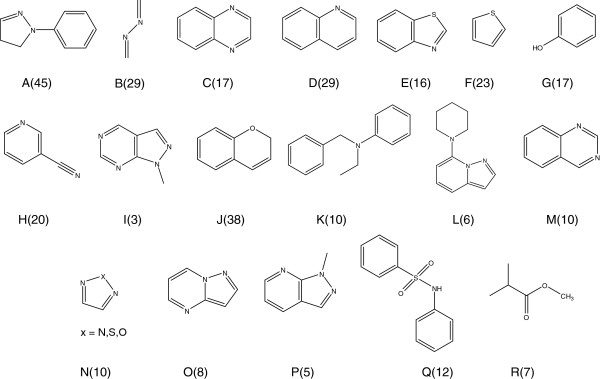
**Structures of 18 BASs obtained for procaspase-3 activators.** The numbers in parentheses show the number of active compounds covered by the associated BAS**.**

The last target was procaspase-3 activators in the PubChem BioAssay database (AID: 463141). There are 326,136 compounds in the database, of which only 350 exhibit the desired activity. In total, 459 rules were obtained from three sampling datasets, of which 86 rules were selected by rule priority. Further refinement and unification resulted in the 13 BASs (A to M) shown in Figure 15. These BASs supported 66.3% of the active compounds. This low coverage is likely the result of the structural diversity of the compound library. An experienced chemist surveyed the structures of 118 of the remaining active compounds and identified five additional BASs shown by N, O, P, Q, and R in Figure 15, which covered additional 18 active compounds.

## Conclusions

BASs of three pharmacological activities were successfully mined from large-scale databases, including real HTS data in the PubChem BioAssay database. Most BASs could be mined without *a priori* chemical knowledge, and the time required to perform the mining for one activity was about 3 days. All of the software developed is open to the public and is available at the BASiC website. The obtained BASs were deemed meaningful substructures from the viewpoint of a medicinal chemist. Thus, BAS mining was shown to be a useful method for understanding the characteristics of active structures. Currently, the bottleneck of this mining process is the unification of similar BASs, which requires a manual comparison of about 100 candidate BAS structures. Future developments to the current process will therefore include an automatic method to identify similar candidate BASs and to construct new, refined BASs. Software development to mine BASs with fewer covered compounds is also in progress.

## Abbreviations

BAS: Basic active structure; BSS: Between-groups sum of squares

## Competing interests

The authors declare that they have no competing interests.

## Authors’ contributions

NT developed KNIME workflows and carried out the mining task, NO developed the refinement software, and TO planned and participated in all aspects of the work. All authors read and approved the final manuscript.

## Supplementary Material

Additional file 1 Contains a brief introduction to the cascade model. Click here for file

Additional file 2 Illustrates how BAS candidates were converted to a unified BAS. Click here for file

## References

[B1] Okada T The development of a knowledge base for basic active structures: an example case of dopamine agonists Chemistry Central Journal 2010 4 1 10.1186/1752-153X-4-1 20181001 PMC2829562

[B2] BASiC [http://BASiC.dm-lab.info/]

[B3] PubChem [http://pubchem.ncbi.nlm.nih.gov/]

[B4] Schierz AC Virtual screening of bioassay data Journal of Cheminformatics 2009 1 21 10.1186/1758-2946-1-21 20150999 PMC2820499

[B5] Qingliang L A novel method for mining highly imbalanced high-throughput screening data in PubChem Bioinformatics 2009 25 24 3310 3316 10.1093/bioinformatics/btp589 19825798 PMC2788930

[B6] Koyama M Hasegawa K Arakawa M Funatsu K Application of rough set theory to high throughput screening data for rational selection of lead compounds Chem-Bio Informatics Journal 2008 8 3 85 95 10.1273/cbij.8.85

[B7] Hasegawa K Migita K Funatsu K Visualization of molecular selectivity and structure generation for selective dopamine inhibitors Molecular Informatics 2010 29 11 793 800 10.1002/minf.201000096 27464269

[B8] Berthold MR Preisach C, Burkhardt H KNIME: the Konstanz information miner Data analysis, machine learning and applications 2008 Berlin, Heidelberg: Springer-Verlag 319 326

[B9] Accelrys: MDDR [http://accelrys.com/products/databases/bioactivity/mddr.html]

[B10] OpenEye [http://www.eyesopen.com/quacpac]

[B11] Pommier Y Integrase inhibitors to treat HIV/AIDS Nat Rev Drug Discov 2005 4 236 248 10.1038/nrd1660 15729361

