# Supplementary material for: Mining basic active structures from a large-scale database
Source: J Cheminform. 2013 Mar 16;5:15. doi: 10.1186/1758-2946-5-15 (PMC3618305; doi:10.1186/1758-2946-5-15)
Supplement: Additional file 1 — Contains a brief introduction to the cascade model. [file 1758-2946-5-15-S1.pdf]

Introduction to the Cascade Model

The cascade model is a mining method for generating characteristic rules [1,2]. It detects links in the itemset lattice where the activity ratio changes sharply in many compounds. Figure 1 shows a typical example of a link and its rule expression. Here, the problem contains four explanatory variables—OH, CO, Me, COO—and a target variable acid, which take (*y*, *n*) values. The itemset at the upper end of the link contains item [OH: *y*], and another item, [CO: *y*], is added along the link. The two small tables at the side of the nodes show the frequencies of the items. We can see that a large distribution change in acid (60/40 to 54/6) occurs with addition of the [CO: y] item. The distribution of COO also changes sharply. The cascade model searches for such links with large distribution changes, and expresses them as rules.

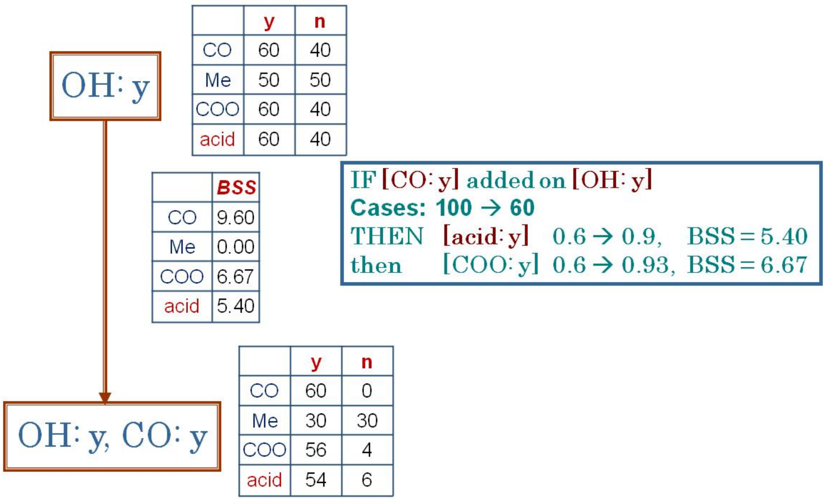

Figure 1 Rule by the cascade model.

The textbox at the right in Figure 1 shows the derived rule. The added item [CO: y] appears as the main condition of the rule, while the items on the upper node ([OH: y] in this case) are preconditions. The acid distributions before and after

application of the main condition are shown in the THEN clause as well as [COO: y] distributions denoted in a then clause at the bottom line.

The cascade model employs the between-groups sum of squares (BSS) as a measure of rule strength to select these characteristic rules. This BSS is also used in the refinement process. Equation (1) is the sum of squares definition for categorical data as given by Gini [3]. It is a simple extension of the numerical sum of squares concept.

$$TSS = \frac{n}{2}\left(1 - \sum_i p_i^2\right) \qquad (1)$$

$$WSS^g = \frac{n^g}{2}\left(1 - \sum_i {p_i^g}^2\right) \qquad (2)$$

$$BSS^g = \frac{n^g}{2}\sum_i \left(p_i^g - p_i\right)^2 \qquad (3)$$

$$TSS = \sum_g \left(WSS^g + BSS^g\right) \qquad (4)$$

The total sum of squares (TSS) can be decomposed into the within-group sum of squares (WSS) and BSS contributions, as shown in Equations (2)-(4), where n and p_i denote the number of cases and the probability of taking value i, respectively, and the superscript g is attached to designate the group.

The tree in Figure 2 shows an example of TSS decomposition. The top of the tree represents 1000 cases consisting of 800 positives and 200 negatives. The corresponding TSS value is 160, as calculated by Equation (1). The cases are then divided into two groups at the bottom according to some criterion. The distributions in these groups are 760/40 on the lower left and 40/160 on the lower right, with BSS values of 18 and 72, respectively. The distribution in the right group is inversely related to that at the top of the tree. Hence, the right link has a higher BSS value despite the

smaller number of cases. We can also see that the equality of Equation (4) holds for this sample.

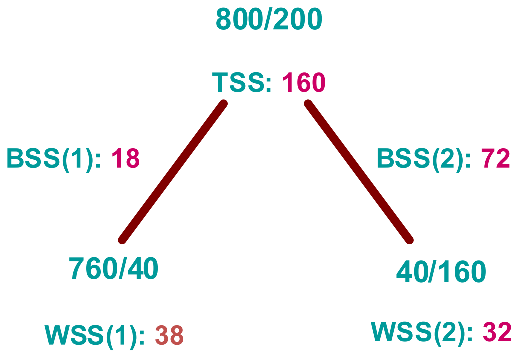

Figure 2 A tree showing an example of TSS decomposition into WSS and BSS.

References

1. Okada T: **Rule Induction in Cascade Model based on Sum of Squares Decomposition.** In *Principles of Data Mining and Knowledge Discovery, PKDD'99. Volume 1704*. Edited by Djamel A, Zighed J, Komorowski J, Żytkow M. Springer; Berlin; 1999:468-474. [*Lecture Notes in Artificial Intelligence*]

2. Okada T: **Efficient Detection of Local Interactions in the Cascade Model.** In *Knowledge Discovery and Data Mining, Current Issues and New Applications, PAKDD-2000. Volume 1805*. Edited by Terano T, Liu H, Chen ALP. Springer; Berlin; 2000:193-203. [*Lecture Notes in Artificial Intelligence*]

3. Gini CW: ***Studi Economico-Giuridici della R. Universita de Cagliari*** 1912. This article is reviewed in Light RJ, Margolin BH: **An analysis of variance for categorical data.** *J Amer Stat Assoc* 1971, 66:534-544.
