# Supplementary material for: Mining basic active structures from a large-scale database
Source: J Cheminform. 2013 Mar 16;5:15. doi: 10.1186/1758-2946-5-15 (PMC3618305; doi:10.1186/1758-2946-5-15)
Supplement: Additional file 2 — Illustrates how BAS candidates were converted to a unified BAS. [file 1758-2946-5-15-S2.pdf]

# Unification of BAS candidates with similar structures

First, BAS candidates with similar structural characteristics were identified. Three candidate structures are shown in the example in Figure 12. Structures are given as SMARTS strings. The numbers of supporting compounds are shown in the following table, where diagonal cells from row A and column A' show the numbers of compounds supported by A_notA’, A_and_A', and by notA_A', respectively. In this case, the compounds supported by A' and A" were all covered by A. Therefore, the generalized structure A was selected as the BAS. Generally, an analyst would see the number of compounds in the table and change the SMARTS expression of the BAS to obtain a satisfactory result.

SMARTS expressions and their structures for BAS candidates

BAS_A:

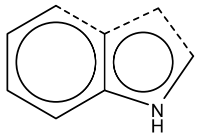


[c;X3;H0]:[c;X3;H0](:c(:c(:c(:c)))):[n;X3;!H0](:c)

BAS_A':

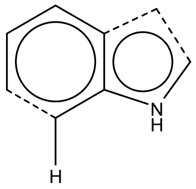


[c;X3;!H0]:[c;X3;H0](:c(:c(:c(:c)))):[n;X3;!H0](:c)

BAS_A":

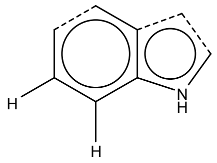


[c;X3;!H0](:c):[c;X3;!H0]:[c;X3;H0](:c(:c)):[n;X3;!H0](:c)

Table. Numbers of supporting compounds by candidate structures.

| | BAS-A | BAS-A’ | BAS-A’’ |
|---|---|---|---|
| BAS-A | #A : 38 | #A_notA’: 4<br>#A_A’: 34<br>#notA_A’: 0 | #A_notA’’: 5<br>#A_A’’: 33<br>#notA_A’’: 0 |
| BAS-A’ | | #A’: 34 | #A’_notA’’: 1<br>#A’_A’’: 33<br>#notA’_A’’: 0 |
| BAS-A’’ | | | #A’’: 33 |
